# A Regional Analysis of Hepatitis C Virus Collaborative Care With Pharmacists in Indian Health Service Facilities

**DOI:** 10.1177/2150132718807520

**Published:** 2018-10-22

**Authors:** Rebecca Geiger, Jessica Steinert, Grant McElwee, Jennifer Carver, Robert Montanez, Julie Niewoehner, Cassandra Clark, Brigg Reilley

**Affiliations:** 1 Indian Health Service, Oklahoma City Administrative Area, Claremore, OK, USA; 2 Indian Health Service, Lawton, OK, USA; 3 Indian Health Service, Pawnee, OK, USA; 4 Indian Health Service, White Cloud, KS, USA; 5 Indian Health Service, Wewoka, OK, USA; 6 Indian Health Service, Haskell, KS, USA; 7 Indian Health Service, Clinton, OK, USA; 8 Northwest Portland Area Indian Health Board, Portland, OR

**Keywords:** hepatitis C virus, access to care, rural, American Indian/Alaska Native

## Abstract

**Background:** American Indian/Alaska Natives (AI/ANs) are disproportionately affected by hepatitis C virus (HCV), with more than double the national rate of HCV-related mortality as well as the highest rates of acute HCV. The “cascade of care” for HCV consists of screening, confirmation, treatment, and sustained virologic clearance (SVR)/cure. At each stage of this process, patients can be lost to follow-up. Federal health care facilities in an administrative area of the Indian Health Service conducted a review to identify and address gaps in HCV treatment. Facilities generally treated HCV with a strong pharmacy component using a collaborative practice agreement and HCV telehealth services to external specialists. **Methods:** All facilities had a pharmacist HCV program point of contact. Each pharmacist conducted a chart review of HCV patients and submitted aggregate results on HCV antibody status, HCV confirmation testing, stage of liver disease, initiation of treatment, and SVR/cure. Each facility also ranked current barriers to scaling up HCV treatment services from a defined list of options. **Results:** Of 1789 HCV antibody positive patients, 77% (1381) had a confirmation test, of which 67% (929) were positive. Of these patients, 62% (576) had their liver fibrosis scored, and 58% (335) had initiated treatment. Of patients with an SVR/cure test, all (274/274) were negative. **Discussion:** These data indicate that rural clinics can be successful providing HCV diagnosis and treatment. Pharmacists can play a key role in HCV clinical services. The outcomes of each step in the treatment process at the facility level can vary widely due to local factors. The barriers to HCV care that persist are nonclinical.

## Background

Approximately 3.5 million persons in the United States have chronic infection with hepatitis C virus (HCV), and about half are unaware of their infection.^1^ Although HCV can be asymptomatic for decades, it is a public health priority: HCV is the leading cause of liver cancer and liver transplants, and it causes more deaths each year in the United States than all reportable infectious diseases combined.^2^

A cure has a tremendous impact on patient prognosis. Among HCV-infected persons, sustained virologic response (SVR) is associated with a >70% reduction in the risk of hepatocellular carcinoma, and a 90% reduction in the risk of liver-related mortality and liver transplantation.^3-5^

The simplified treatment regimens have allowed more HCV treatment to be performed at the primary care level.^6^ Telehealth programs have proven successful in supporting HCV treatment by primary care clinicians.^7^

American Indian/Alaska Natives (AI/ANs) are disproportionately affected by HCV, with more than double the national rate of HCV-related mortality as well as the highest rates of acute HCV.^8^ In addition, Oklahoma has the highest seroprevalence of HCV in the nation at 3.34%.^9^ The Indian Health Service (IHS) is the leading provider of care to AI/AN communities, serving an estimated 2.2 million persons, often in rural primary care health facilities.^10^

There are 6 federal IHS “Service Units” (SUs) composed of one or more health facilities in Oklahoma City Area (comprising Oklahoma, Kansas, Texas), of which 9 are in Oklahoma and 2 in Kansas. To adapt to human resource shortages,^11,12^ many facilities enhanced HCV clinical capacity with practice collaborative agreements with pharmacists. As medication experts with training in HCV disease state management, clinical pharmacists are in a unique position to increase access to care and improve health outcomes for AI/AN patients with an HCV diagnosis. Collaborative practice agreements outline the clinical pharmacists’ responsibilities to provide comprehensive care to HCV patients under the supervision of a physician. Such agreements allow pharmacists to place laboratory orders, determine medication regimens and duration of therapy, manage medication procurement and manage side effects. In addition, pharmacists provide detailed medication counseling and identify prescription and over-the-counter drug interactions to increase treatment adherence and likelihood of HCV cure. To provide comprehensive HCV treatment, pharmacists order labs and interpret their results; screen for and address lifestyle factors and comorbidities that may adversely affect HCV treatment outcomes. A clinical pharmacist in this setting may act as a case manager for the patient diagnosed with HCV, linking the patient to other services.

Regional and local leadership have sought to make HCV treatment more accessible for clinicians and patients at the primary care level. To meet HCV treatment coverage requirements for a specialist consultation for HCV patients, the Oklahoma City Area has negotiated a waiver with Oklahoma Health Care Authority on a facility-by-facility basis, contingent on the level of HCV treatment experience level within the facility. In addition, clinicians can use national and regional telehealth options for specialist support.

The multiple steps in HCV diagnosis and treatment or “cascade of care” for HCV includes screening, RNA confirmation, treatment, and cure (sustained virologic response or SVR12, defined as undetectable viral load, 12 or more weeks after completion of treatment). At each stage of this process, patients can be lost to follow-up.^13^

Federal facilities in the Oklahoma City Area conducted a review to identify and address gaps in certain key steps in HCV treatment. Collectively, these facilities have an active clinical population (defined as 2 or more medical visits in the past 3 years) of about 170 000 tribally enrolled patients.

## Methods

At each of the 6 federal SUs, investigators compiled data comprising 11 separate health facilities in Oklahoma and Kansas. Each investigator was a pharmacist with an integral role in HCV treatment. These facilities range in size from a large hospital with more than 100 000 active clinical patients to a small clinic with a few hundred.

Each investigator compiled aggregate data on HCV patient status from a standardized federal electronic health record. Clinical variables included antibody and viral load/RNA testing, fibrosis according to AST (aspartate aminotransferase) to platelet ratio index (APRI) or fibrosis-4 calculations,^14^ initiation of treatment, completion of treatment, and SVR 12 weeks postcompletion of treatment. Tribal facilities were not included due to data sharing considerations.

All federal facilities use the same health information platform and electronic health record. Patient records were individually reviewed to determine patient laboratory results and treatment status. Data on HCV was inclusive of all known historical HCV data (from the implementation of the IHS electronic health record in the early 2000s, although with some variation by facility) through 2017. These data were reviewed by the Oklahoma City Area Institutional Review Board and deemed exempt as nonresearch.

## Results

**Figure 1.**
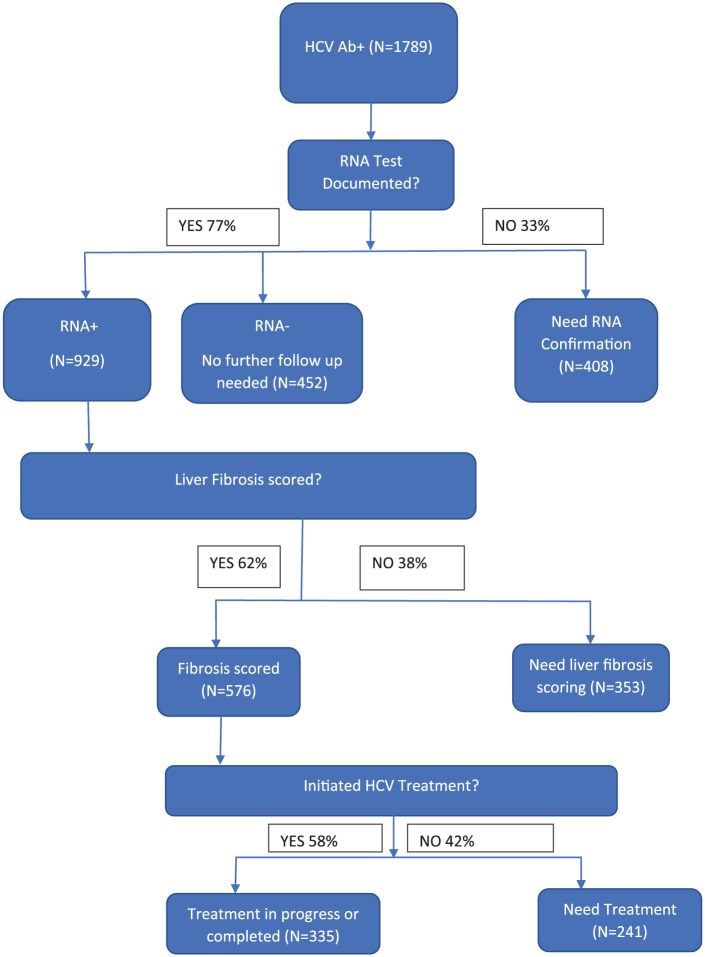
Hepatitis C virus (HCV) patient diagnoses and treatment (cumulative), Oklahoma Administrative Area federal health facilities, Indian Health Service, December 2017.

Overall, the facilities reported 1789 patients with HCV+ antibody (range 16-800), of whom 335 (18.7%) had initiated or completed treatment. The study documented the keys step in diagnosis and treatment, including RNA confirmation, scoring of liver fibrosis, and initiation of treatment (Figure 1). Patients with an RNA confirmation test documented, but not with an RNA+ results, are presumed to have had spontaneous clearance of HCV, and do not represent a point for further clinical follow-up. The step with the greatest proportion of patients needing following up occurred where patients were ready to treat (liver fibrosis staged) to initiation of treatment.

There were variations by facility at each step of the HCV treatment process. These wide ranges encompassed all measures: RNA test documented (62.1%-100%), proportion of tested patients with RNA+ result (56.6%-89.8%), fibrosis scoring done (0%-96.8%) and initiated or completed treatment (28.6%-57.9%).

Of patients who had liver fibrosis scoring performed, 23.7% (137/576) had stage F3 (advanced fibrosis) or higher. A total of 28 patients were currently in treatment (range 0-8). All patients with who had completed treatment and had an SVR12 test were negative (247/247). Overall, this represents 26.5% of patients with confirmed HCV RNA (247/929) or 18.5% of HCV patients with confirmed chronic HCV and undocumented RNA status (247/1337).

## Limitations

These data did not seek to track default or treatment failures, and likely overestimates true SVR rates as complicated patients may be referred to external specialists, although treatment outcomes are still thought to be excellent. These data likely overrepresent historical rather than recent infection due to screening of baby boomers and underrepresents HCV among younger patients associated with the nationwide opioid epidemic. These data do not include key variables such as age, sex, or residence, which would enable analysis of the HCV patient cohort and identifying what may be associated with patients progressing to initiation and completion of treatment. Finally, IHS is dependent on third party payers and patient assistance programs for medications, so drug access in this region may differ substantially compared with other IHS regions.^15,16^

## Discussion

These data indicate that rural clinics using collaborative practice agreements with pharmacists can be instrumental in HCV services at the primary care level and have strong outcomes in HCV treatment/SVR12. These results also identify important gaps that persist at the facility and regional level; a majority of confirmed HCV patients still need treatment. The greatest attrition in the HCV “cascade of care” that need clinical follow-up include relinking patients to care for RNA test confirmation, scoring patient liver fibrosis, and initiating care.

All facilities are believed to have had access to similar interventions for policy and practice (electronic clinical decision reminder for screening, HCV clinical training, HCV patient paneling software, and telehealth support for treatment), but there is variability in capacity to implement, which are believed to be linked to time/human resources and competing priorities rather than technical constraints.

Further research is needed on key questions, such as what barriers keep patients from re-linking to care in terms of transport, stigma, perceived costs or efficacy of treatment. Similarly, the high proportion of Ab+ with a negative RNA+ in some facilities bears further investigation into which patients have spontaneously cleared the virus.
